# A Comparative Analysis of the Water Retention Properties of Hydrogels Prepared from Melon and Orange Peels in Soils

**DOI:** 10.3390/gels11010008

**Published:** 2024-12-27

**Authors:** Shiwei Fang, Yuan Zhong, Jun Wu, Yufan Xie, Liqun Cai, Minjun Li, Jun Cao, Hejie Zhao, Bo Dong

**Affiliations:** 1 College of Resources and Environmental Sciences, Gansu Agricultural University, Lanzhou 730070, China; 13892801256@163.com (S.F.); 18809453467@163.com (Y.X.); cailq@gsau.edu.cn (L.C.); l1174219037m@163.com (M.L.); dongbobby@163.com (B.D.); 2 State Key Laboratory of Aridland Crop Science, Gansu Agricultural University, Lanzhou 730070, China; zhongy@gsau.edu.cn; 3 Research Center for Water-Saving Agriculture in Gansu Province, Lanzhou 730070, China; 4 Agricultural Technical Extension Station of Gannan Tibetan Autonomous Prefecture, Gannan 747000, China; 18109411990@163.com (J.C.); 13321240991@163.com (H.Z.)

**Keywords:** hydrogel, melon peel, orange peel, radical polymerization, water retention, drought stress

## Abstract

The objective of this study was to conduct a comparative analysis of the performance of hydrogels prepared from two distinct raw materials and to identify the hydrogels with the optimal overall capacity for dry farming applications. Ten grafted polymer hydrogels were prepared from melon peel (MP) and orange peel (OP). A comparative analysis of the degree of swelling, water absorption time, pH range, reusability, and soil water retention and water-holding capacity of the two hydrogels revealed that the MP-based hydrogels exhibited superior performance in all evaluated parameters when compared to their OP-based counterparts. The treatment group of hydrogels prepared from MPs exhibited the highest degree of swelling, with an absorptive capacity of up to 765.6 g/g in ultrapure water. The optimum absorption ratio at pH = 8.1 was 606.8 g/g, as determined by Gaussian distribution modeling. The treatment group with the best reusability demonstrated an average absorption ratio of 445.0 g/g. The degree of swelling was 84.0 g/g when the process was repeated seven times. After the MP-gels were applied to the soil, it was observed that the gels enhanced the water retention and holding capacity of the sandy soil. The water retention ratio of the sandy soil was increased by 271.0% by the addition of MP-gel, and the growth of wheat was found to be normal when 1.5% to 2.0% of MP-gel was added under drought-stress conditions. In light of the necessity to reuse agricultural waste, the preparation of MP-gel can facilitate the improvement of dry farming and address the issue of water scarcity in agriculture. This offers a viable solution for the growth and management of crops under conditions of drought stress.

## 1. Introduction

The substantial impact of drought on agricultural production results in considerable socio-economic losses, particularly given that prolonged drought stress disrupts crop metabolism, affects normal crop growth and development, and may even lead to crop extinction [1]. In the context of prolonged drought, a notable limitation on crop growth is the decline in soil water retention and water-holding capacity [2]. A reduction in water availability will impact the structure and activity of soil microbial communities, as well as the supply of essential soil nutrients [3]. This will result in a reduction in crop absorption of nitrogen, phosphorus, and other nutrients, ultimately hindering crop growth. In light of the challenges posed by climate change, it is imperative to enhance crop resilience to drought stress and optimize soil water retention to ensure sustainable agricultural development [4]. The implementation of water-absorbing materials that function as soil reservoirs can facilitate the attainment of enhanced water-use efficiency in dry agricultural settings. These materials enable the prolonged re tention of water within the soil, thereby enabling crops to access a more sustained supply of water.

In a recent study, Zhang et al. [5] developed a new biodegradable soil water retention agent synthesized by microwave radiation technology based on polyaspartic ammonia and bentonite. This agent exhibits high water absorption properties and good degradation ratios, making it a promising alternative for use in soil conditioning and water retention. This research trend reflects the urgent need among scholars to identify more environmentally friendly raw materials. In this context, Chen et al. [6] synthesized an environmentally friendly hydrogel composed of borax and acacia bean gum, with the dual objective of enhancing the water retention capacity of sandy soil and realizing the resourceful use of waste materials. Duong et al. [7]. investigated a cellulose-chitosan aerogel based on agricultural waste, which has a wide range of applications due to its biocompatibility, biodegradability, and antimicrobial ability. Furthermore, Xu et al. [8]. developed a biocarbon material that was co-modified with KOH and chitosan, exhibiting high chitosan loading, uniform distribution, and excellent adsorption efficiency. The potential of starch-blended biodegradable polymers in realizing eco-friendly innovations is highlighted in the study of Singh et al. [9]. These materials not only reduce environmental pollution but also promote a circular economy. Indeed, there is an emerging trend in the utilization of agricultural waste materials, including fruit peels, tailings, biochar, and biomass energy raw materials, for the production of natural superabsorbent hydrogels. Agricultural waste materials contain polymeric hydrophilic substances, including sugars and cellulose, which fill the organic matter of the gel and provide water absorption. This not only provides a secondary use for the waste material but also renders the material itself non-toxic and harmless, thus preventing soil contamination by organic matter.

Melon and orange peels, which are ubiquitous byproducts of fruit processing, are frequently disregarded. However, they are rich in macromolecules such as cellulose, pectin, and vitamin C [10], rendering them ideal raw materials for the preparation of hydrogels. In a study by Tabrez et al. [11], melon peels were successfully converted into biocarbons with excellent water-absorbing properties and the potential for reuse. Sarkari et al. [12] developed a water absorbent in aqueous solution by using wheat straw and orange peel, resulting in an absorbent that exhibits high water absorption. These studies demonstrate the significant potential of melon and orange peels for hydrogel applications. Nevertheless, these two materials have rarely been employed in the synthesis of natural superabsorbent hydrogels for application in dry agriculture. The objective of this study was to examine the impact of superabsorbent hydrogels derived from melon and orange peels on soil water retention. It is noteworthy that, despite certain similarities in their properties, melon rinds and orange peels also exhibit notable differences. Melon peels have been found to contain higher levels of water, polysaccharides, and pectin, while orange peels have been observed to have relatively lower levels of water and pectin, but higher levels of cellulose, lignin, dietary fiber, and vitamins [13].

In this study, we synthesized melon peel-acrylic acid/acrylamide superabsorbent hydrogels (MP-AA-AAM-gels) and orange peel-acrylic acid/acrylamide superabsorbent hydrogels (OP-AA-AAM-gels) by grafting fresh melon peels and orange peels, respectively, onto copolymerized monomers, and used an acrylic acid/acrylamide hydrogel without added peel waste (CK) as a control. This study aimed to investigate and comparatively screen the physical properties, swelling capacity, and reusability of the two hydrogel materials in order to select the best material for soil water retention tests and to promote the growth of wheat seedlings under drought-stress conditions.

## 2. Results and Discussion

### 2.1. FTIR Spectra Characterization Analysis

**Figure 1:**
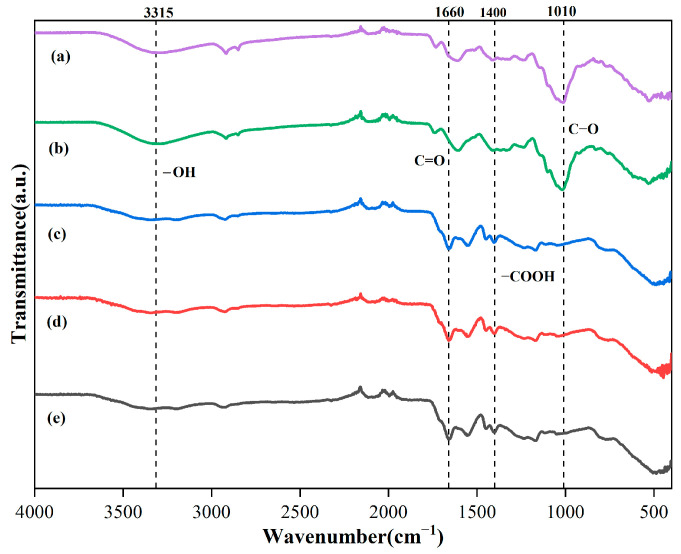
FTIR spectra characterization of (a) MP, (b) OP, (c) MP-gel, (d) OP-gel, and (e) CK.

As shown in Figure 1. During the synthesis of the hydrogel, the absorption peak located at 1660 cm^−1^ is indicative of the vibrational stretching behavior of the C=O functional group. Meanwhile, the absorption. peak at 1540 cm^−1^, which originates from the bending vibration of the C-H bond in the amide group, was observed in both the waste and hydrogel samples. The position of the C-H absorption peak of the amide group exhibited a notable shift at the conclusion of the reaction process. Furthermore, the absorption peak at 1010 cm^−1^, which originally corresponded to the C-O stretching vibration of the hydroxyl group of the alcohol, exhibited a near-complete disappearance during the re-reaction process. This transformation indicates that during the synthesis of the hydrogel, MBA functions as a cross-linking agent, and APS acts as an initiator. The glycosidic bonds in the two waste materials are broken under the influence of APS, resulting in the generation of large molecular radicals. These radicals facilitate the grafting of acrylic acid and acrylamide onto the ends of the broken glycosidic bonds.

**Figure 2:**
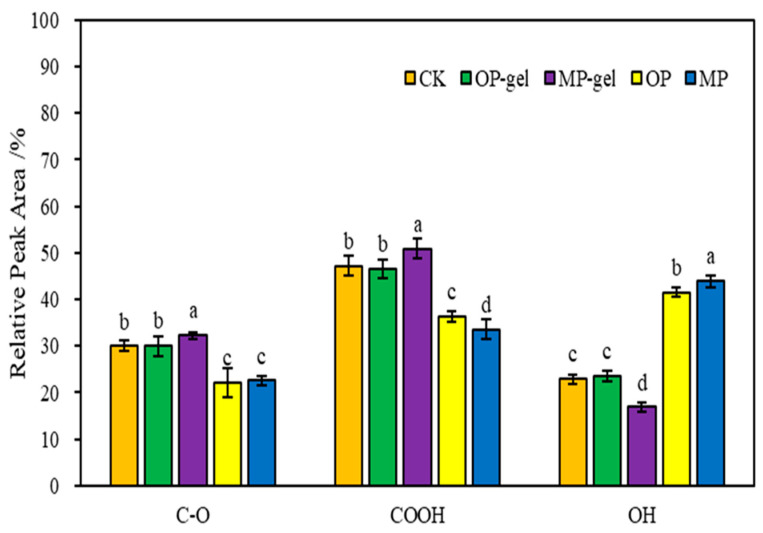
Relative FT-IR peak areas of MP, OP, MP-gel, OP-gel, and CK samples. In the bar chart, the letters (a–d) indicate the level of significant difference between the groups represented by each bar, where “a” means the group with the highest level of significance, followed by “b”, ‘c’ and ‘d’, which represent groups with successively lower levels of significance.

The relative peak areas of each material are shown in Figure 2. The relative peak area of the C-O bond in CK was 29.94%, which was employed as a baseline for comparison. Following modification, the relative peak area of C-O bonds in OP-gel exhibited a slight increase to 29.95%, indicating that the structural alteration of C-O bonds is minimal. In contrast, the relative peak area of C-O bonds in MP-gel exhibited a notable increase, reaching 32.23%. For the carboxyl (COOH) functional group, CK has the largest relative peak area of 47.14%. This finding reflects the presence of a considerable number of carboxyl groups in the original material. Following modification, the relative peak area of the COOH group in OP-gel exhibited a slight decrease, reaching 46.49%. In contrast, the relative peak area of COOH groups in MP-gel increased significantly, reaching 50.91%. In both the MP and OP samples, the relative peak areas of the hydroxyl groups were observed to be larger. However, in the characterization of MP-gel and OP-gel, the relative peak area of the hydroxyl group was markedly diminished in comparison to that of MP and OP. This observation suggests that the hydroxyl group was engaged in a chemical reaction during the synthesis process, which resulted in a reduction in its content.

The detailed analysis of infrared spectra in this study demonstrates that the molecular structure of the matrix undergoes a significant transformation during the chemical modification process. Specifically, the appearance of the stretching vibrational absorption peaks of the N-H bond not only confirmed the successful introduction of acrylamide but also indicated the stability of the amide bond in the modified material [14]. This demonstrated that the modification process was effective in regulating the structure of the material. Concomitantly, the substantial intensification of the stretching vibrational peaks of the C=O bond substantiates the incorporation of acrylic acid and the unimpeded progression of the esterification reaction. This finding serves to further confirm the conversion of functional groups and the remodeling of the molecular structure during the chemical modification process. The alterations in the frequencies and intensities of the amide and carboxylic acid functional group absorption peaks offer insights into the dynamic trend of these functional groups throughout the chemical modification process [15]. This changing trend not only reflects the increase or decrease in functional groups, but also reveals the chemical reconfiguration process of the molecular structure. In particular, the displacement of the absorption peak of the amide C-H bond implies a subtle change in the intramolecular environment, which may be caused by intermolecular interactions, the formation of hydrogen bonds, and possibly chemical cross-linking [16]. The disappearance of the absorption peak at 1010 cm^−1^ during chemical modification is an important observation that clearly indicates the change of alcohol hydroxyl groups on the surface of the material. This shift is most likely due to the role of the alcohol hydroxyl groups in the cross-linking reaction, which led to the successful formation of the hydrogel. This finding coincides with the previous results of Gorgieva et al. [17] and further validates the key role of alcohol hydroxyl groups in the cross-linking process.

### 2.2. SEM Characterization Analysis

**Figure 3:**
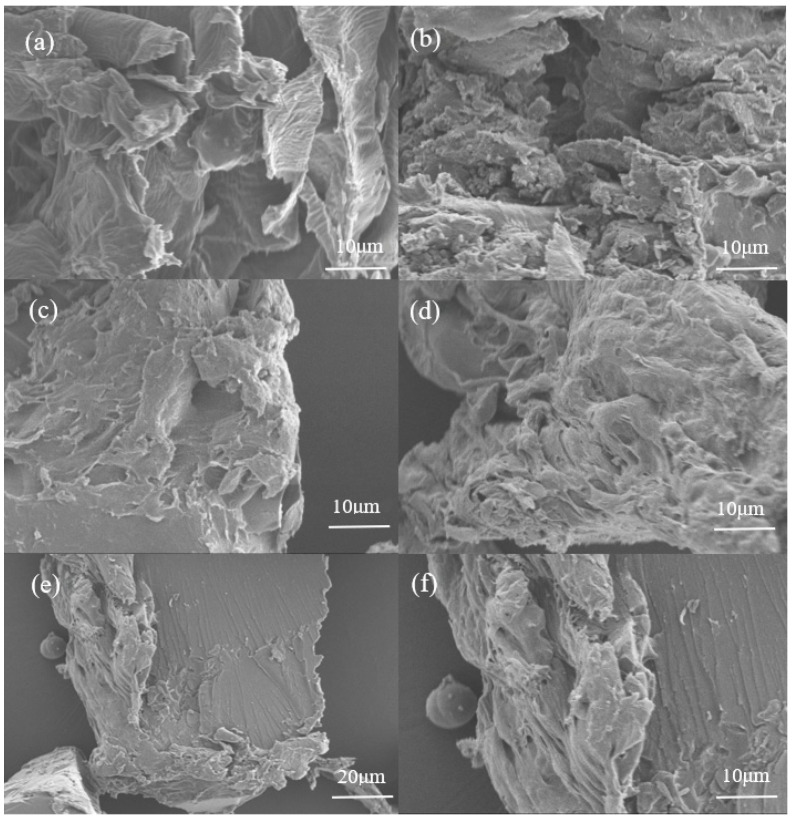
Surface characteristics of (**a**) MP, (**b**) OP, (**c**) MP-gel, (**d**) OP-gel, and (**e**,**f**) CK. (**e**) has a magnification of 1.0 k, while (**a**–**d**,**f**) have a magnification of 2.0 k. The working distance of the microscope is 6.5 mm for all images.

Figure 3 illustrates that the microstructures of MP-gel and OP-gel are markedly distinct from those of MP and OP. The surfaces of MP-gel and OP-gel exhibit a furrowed and porous texture, with interconnected networks formed between the pores, and the surface topology displays a rough texture. In contrast, the surfaces of MP and OP are distinguished by a smoother texture and a less intricate pore structure. It is noteworthy that CK displays a network-like structure, yet exhibits a markedly reduced number of pores in comparison to MP-gel and OP-gel.

The SEM analysis revealed that the modification of MP and OP materials led to the formation of a complex three-dimensional network structure, which plays a crucial role in enhancing the water absorption and retention properties of the hydrogels. The chemical modification not only increases the contact area with water molecules but also improves the stability of the network, thereby accelerating the water absorption ratio and enhancing water retention capacity. In particular, the MP- (AA-AAM)- gel exhibited a richness and diversity of pores, which contributed to a significantly higher number of surface pores compared to the OP- (AA-AAM)- gel. This structural distinction is a key factor in the superior water uptake and retention capabilities observed in the MP-gel. The results align well with the findings of Su et al. [18], who demonstrated the impact of raw material composition and modification on the performance of superabsorbent polymers, emphasizing the importance of material design in achieving optimal water retention properties in hydrogels. The structural differences highlighted by the SEM analysis suggest that the choice of raw materials and the modification process are critical in determining the hydrogel’s performance.

### 2.3. Analysis of Water Absorption of Hydrogel

**Figure 4:**
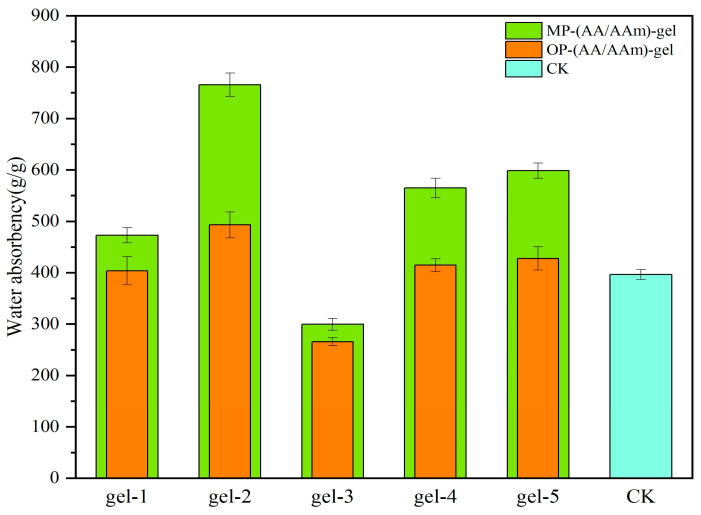
Water absorption of MP-gel, OP-gel, and CK samples.

Figure 4 illustrates the water absorption of the MP-gel, OP-gel, and CK at their respective dissolution equilibrium points. When the monomer ratio was fixed and the MBA addition was varied, the water absorption of MP-gel and OP-gel for the gel-2 treatment was the highest among the two materials. The water absorption of MP-gel-2 reached 765.63 g/g, which was significantly higher than that of the other treatment groups. This suggests that a reduction in the quantity of the cross-linking agent results in an enhanced capacity for water absorption by the hydrogel.

Under fixed MBA addition, but with varying ratios of AA to AAM, the water absorption ratios of MP-gel and OP-gel exhibited similar patterns. Gel-5 had the highest water absorption ratios, followed by gel-4, and then gel-1. This indicates that the monomer ratio also affects the hydrogel’s water absorption capacity, but not as significantly as the MBA addition amount.

**Figure 5:**
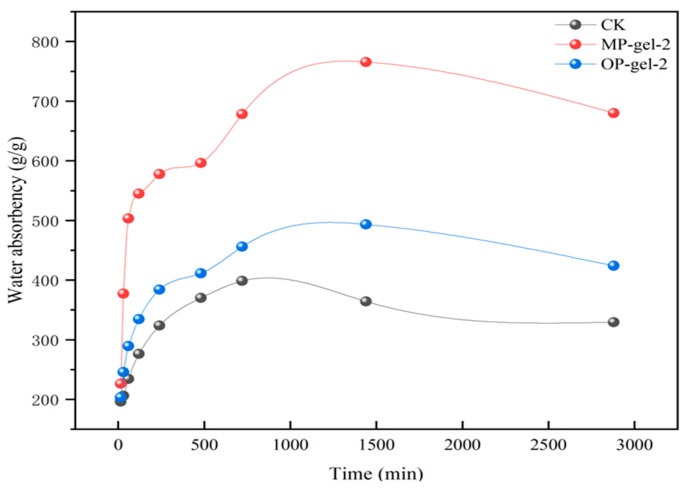
Water absorption of MP-gel, OP-gel, and CK at different times.

In MP-gel and OP-gel, the treatment group gel-2, which exhibited the highest water absorption, was selected for the water absorption time test. As depicted in Figure 5, the CK reached adsorption equilibrium at 720 min, whereas MP-gel-2 and OP-gel-2 achieved their respective swelling equilibria at 1440 min. At 15 min, the water absorption ratios of MP-gel-2, OP-gel-2, and CK were comparable, all around 226 g/g. By 30 min, the water absorption ratio of MP-gel-2 (377.2 g/g) was significantly higher than that of the other two treatments. Across all time points, MP-gel-2 consistently exhibited a higher swelling ratio than OP-gel-2 and CK. Moreover, the water absorption ratio of CK remained lower than that of the other two treatment groups at all tested time points, further substantiating the superior water absorption capacity of MP-gel-2.

In the in-depth study of the correlation between the water absorption performance of hydrogels and the amount of cross-linker used in their preparation, a significant phenomenon was observed: reducing the amount of cross-linker can markedly enhance the water absorption capacity of hydrogels. Specifically, under experimental conditions where the monomer ratio is kept constant and only the MBA addition amount is adjusted, the gel-2 treatments of MP-gel and OP-gel demonstrated the highest water absorption ratios, with MP-gel-2 significantly surpassing other experimental groups in terms of water absorption ratio. Further research has revealed that excessive use of MBA can significantly weaken the water absorption capacity of hydrogels. This phenomenon can be attributed to the increase in cross-linking density when the MBA content increases, which in turn hinders the free entry or retention of water molecules within the hydrogel network, leading to a decrease in the water absorption property of the hydrogel. This research finding is in agreement with the work of Kabiri K et al. [19]. In addition, the study by Akhmetzhan et al. [20] on the synthesis of N, N-dimethylacetamide-based hydrogels and their heavy metal adsorption characteristics also provides supporting evidence for this argument, revealing the direct regulatory role of cross-linker concentration on the pore structure and water absorption performance of hydrogels.

MP-gel exhibited a high swelling ratio, which was primarily attributable to the abundant cellulose and pectin present in the MP and its distinctive chemical composition [21]. The presence of cellulose and pectin within the hydrogel results in the formation of a highly compact three-dimensional network structure, which markedly enhances the capacity of the material to adsorb water molecules [22]. The melon rind may also contain components that facilitate the interaction between the hydrogel and water molecules, thereby enhancing the swelling process. These components may include sugar alcohols and specific organic acids [23]. The diminished swelling capacity of hydrogels devoid of supplementary peel was predominantly attributable to the absence of highly absorbent constituents in the fundamental formulation [24]. The absence of additional biomass, such as cellulose and pectin, resulted in a less dense network structure within the gels, which in turn led to a reduction in the number of hydrophilic functional groups. This ultimately constrained their capacity to absorb water. In contrast, the swelling ratio of OP-gel was observed to be slightly higher than that of the CK treatments, yet lower than that of MP-gel. The cellulose and pectin present in the OP provided the fundamental water absorption capacity for the hydrogel. However, the observed lower swelling ratio may be attributed to the inherent characteristics of certain components present in the OP. The high terpenoid content of OP may result in weaker interactions with water molecules under certain conditions, potentially impacting the gel’s water absorption efficiency [25]. Furthermore, the structure and pore characteristics of OP may not be as conducive to water molecule penetration and distribution as those of MP-gel. Consequently, MP is more suitable than OP as a raw material for hydrogel formation by cross-linking polymerization with AA and AAM monomers.

### 2.4. Analysis of Swelling Capacity of Hydrogels at Different pH

**Figure 6:**
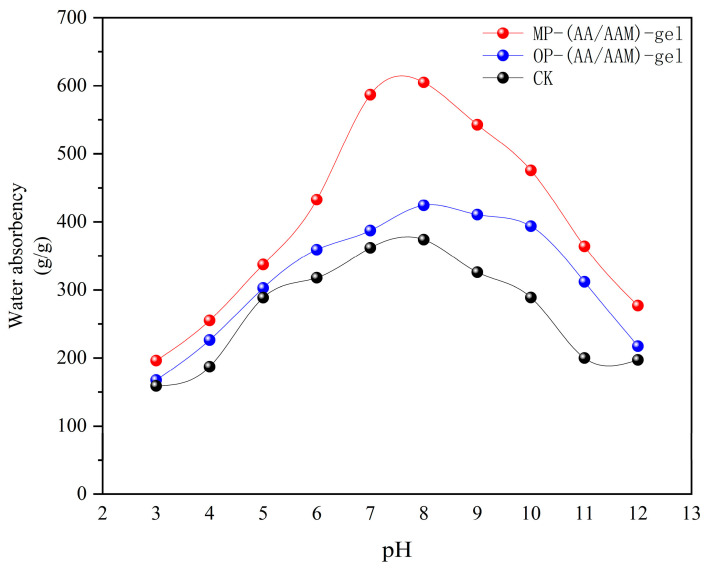
Water absorption of MP-gel, OP-gel, and CK at different pH.

Figure 6 shows the dynamic relationship between the water-absorbing property of MP-gel-2 and pH. It shows that during the incremental increase in pH from 3 to 7, the water-absorbing property of MP-gel-2 rapidly increased and reached an equilibrium state. However, when the pH value continued to rise above 9, its water absorption began to show a gradual decline. The water absorption of OP-gel-2 exhibited a comparable trend to that of MP-gel-2, although the increase was less pronounced. The water absorption of MP-gel-2 was observed to be greater than that of OP-gel-2 and CK at all pH levels.

The water absorption properties of hydrogels are closely related to their chemical structure and the pH of the solution. The water absorption properties of hydrogels in different pH environments are a complex process that is affected by a combination of factors. Zhang et al. [26] have provided us with valuable information that will help us to better understand and utilize hydrogel materials. Li et al. [27] showed that pH-responsive hydrogels have high water absorption and swelling properties, which are related to the dissociable groups in their chemical structure. At neutral pH, these groups are moderately dissociated and are able to effectively form hydrogen bonds with water molecules, resulting in efficient water absorption. The review article by Tang et al. [28] points out that pH-sensitive hydrogels have received a lot of attention due to their water absorption applications. They emphasized the effect of pH on the water absorption performance of hydrogels, especially in hydrogels containing dissociable groups, where changes in pH can lead to changes in the ionization state of the groups, which in turn affects the water absorption ratio. This was further confirmed by Chen et al. [29], who prepared pH-responsive hydrogels that exhibited excellent water absorption properties under specific pH conditions. These findings suggest that the conformation of the polymer chains and the ionization state of the dissociated groups in the hydrogels are the key factors in determining their water absorption properties. Under neutral pH conditions, the polymer chains are in a more relaxed conformation, which facilitates the formation of hydrogen bonds and thus improves water absorption. In contrast, under acidic or alkaline conditions, aggregation or over-swelling of the polymer chains affects the adsorption and retention of water molecules. Therefore, by adjusting the chemical structure and pH value of hydrogels, precise regulation of the water absorption performance of hydrogels can be realized.

**Figure 7:**
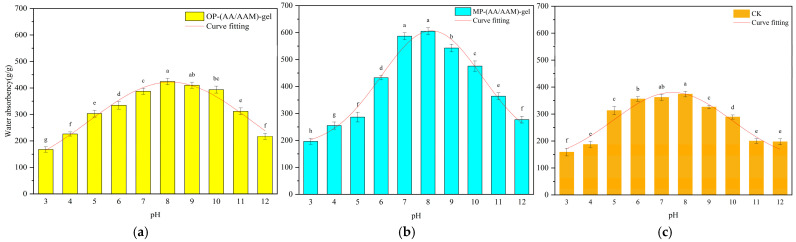
Gaussian distribution fitting curves for (**a**) OP-gel, (**b**) MP-gel, and (**c**) CK. In the bar graphs, the letters (a–g) indicate the level of significant difference between the groups represented by each bar, where “a” indicates the group with the highest level of significant difference, followed by “b”, “c” and “d” until “g”, which denotes the groups with sequentially lower levels of significant difference.

Figure 7 illustrates the variability of water absorption of different hydrogels at various pH values, with Gaussian distribution curves. At pH = 7 and pH = 8, MP-gel and CK exhibit significantly higher absorption than the other pH values. Similarly, at pH = 8 and pH = 9, OP-gel and CK demonstrate significantly higher absorption than the other pH values. The analysis of the fitted curves revealed that the amplitude of the MP-gel was significantly larger, while the amplitudes for CK and OP-gel were comparatively smaller. This suggests that the water absorption capacity of the MP hydrogels is more sensitive to changes in pH compared to CK and OP-gel.

A Gaussian distribution model was employed to fit the optimal pH and corresponding optimal water absorption of the three hydrogels. The results indicated that MP-gel-2 and OP-gel-2 exhibited the highest water absorption at pH = 8.14 and pH = 8.11, respectively. The respective values were 606.76 g/g and 421.37 g/g, whereas the CK samples exhibited the highest value of 380.08 g/g for water absorption at pH =7.46.

When investigating the water absorption of hydrogels at different pH values, it was found that the water absorption of hydrogels at different pH showed a normal distribution function, i.e., the water absorption remained high in a neutral environment and decreased in an acidic or alkaline environment. In order to find the optimal pH for the operation of hydrogels, a Gaussian distribution curve was fitted, and the peak of the curve function represented the water absorption of hydrogels at the optimal pH. The peak of the curve function represents the water absorption of the hydrogel at the optimal pH, and the water absorption at the highest point of the fitted curve for the CK material can be carried over to the curves for MP-gel and OP-gel to determine how much the range of applicability of the waste-added hydrogel has been improved in comparison to that of CK, with the water absorption of MP-gel-2 at pH 5.52 and 10.77 exceeding the optimal absorption of CK (based on the CK optimal absorption capacity as the standard). This suggests that the addition of MP has enabled MP-gel to be used in a wider range of pH applications, with a significant reduction in water absorption only occurring in extremely acidic and alkaline environments. This suggests that the hydrogels prepared with the waste material have formed a bilayer network structure that is more resistant to acidic and alkaline environments and stabilizes the distribution of water molecules in the transparent gel, thus allowing it to be used in a wider range of environments.

### 2.5. Reusability of Hydrogels

**Figure 8:**
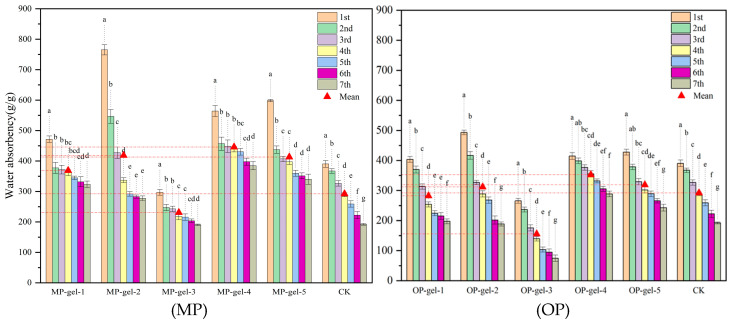
Reusability of MP-gel and OP-gel. The red dotted line represents the value of the vertical coordinate corresponding to the mean value. In the bar graphs, the letters (a–g) indicate the level of significant difference between the groups represented by each bar, where “a” indicates the group with the highest level of significant difference, followed by “b”, “c” and “d” until “g”, which denotes the groups with sequentially lower levels of significant difference.

As shown in Figure 8, the MP hydrogel has a better swelling ability than the OP hydrogel during reuse. In the case of MP, the highest initial swelling ratio of 765.2 g/g was observed for gel-2. However, the swelling ratio decreased significantly with each water uptake and did not reach equilibrium until after the fifth water uptake. This indicates the low reusability of gel-2. In contrast, gel-4 had the most favorable reusability with the smallest decrease in swelling ratio and exhibited significant stability.

Reusability assessment is a key factor in determining the operational life of a hydrogel, as it reflects the hydrogel’s ability to utilize resources sustainably, in line with the principles of a circular economy. In our replicated experiments, MP-gel-2 exhibited a high water absorption ratio during initial use, but the water absorption capacity decreased significantly after several cycles. The underlying reason for this decline may be the low MBA content in MP-gel-2, which leads to structural instability and thus degradation during repeated hydration and dehydration cycles. This finding is consistent with the findings of Lee et al. [30] that hydrogels with low cross-link density are susceptible to structural damage under such conditions, emphasizing the importance of cross-link density in maintaining the structural integrity of hydrogels. In addition, Zhou et al. [31] showed that higher cross-linking densities resulted in higher mechanical strength and stability of hydrogels, which is essential for improving reusability. Therefore, MP-gel-4 employs a more reasonable amount of cross-linker and successfully balances water absorption properties and reusability, providing a more sustainable option for water management applications.

### 2.6. Water Retention and Water-Holding Capacity of Hydrogels in Different Soils

**Figure 9:**
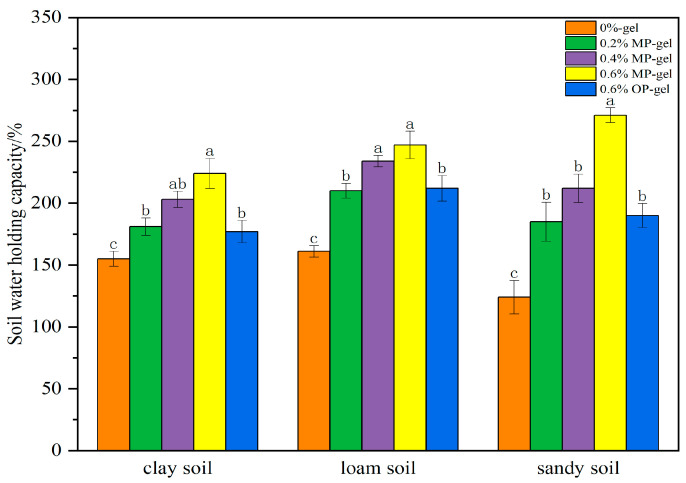
Soil water-holding capacity variations with 0%, 0.2%, 0.4%, 0.6% MP-gel addition in sandy, loam, and clay soils. In the bar graphs, the letters (a–c) indicate the level of significant difference between the groups represented by each bar, where “a” indicates the group with the highest level of significant difference, followed by “b” and “c” which denotes the groups with sequentially lower levels of significant difference.

The effects of different types of hydrogels on the water retention and water-holding capacity of various soil types, including sandy, loamy, and clayey soils, exhibited notable differences. As shown in Figure 9, the water retention effect of the hydrogel varied with soil type, with significant differences between sandy, loamy, and clay soils. The water-holding capacity of the soil was significantly increased by the addition of hydrogel to the sandy soil. The addition of 0.6% hydrogel significantly increased the water-holding capacity from the initial 137% to 271%. In contrast, the clay soil did not improve significantly compared to the sandy soil, increasing from 155% to 224%, and the hydrogel added to the loamy soil had an improvement between the sandy soil and the clay soil, increasing from 161% to 247%. In various soil textures, the water retention capacities of 0.6% OP-gel and 0.2% MP-gel did not differ, which further suggests that MP-gel exhibits superior performance compared to OP-gel.

**Figure 10:**
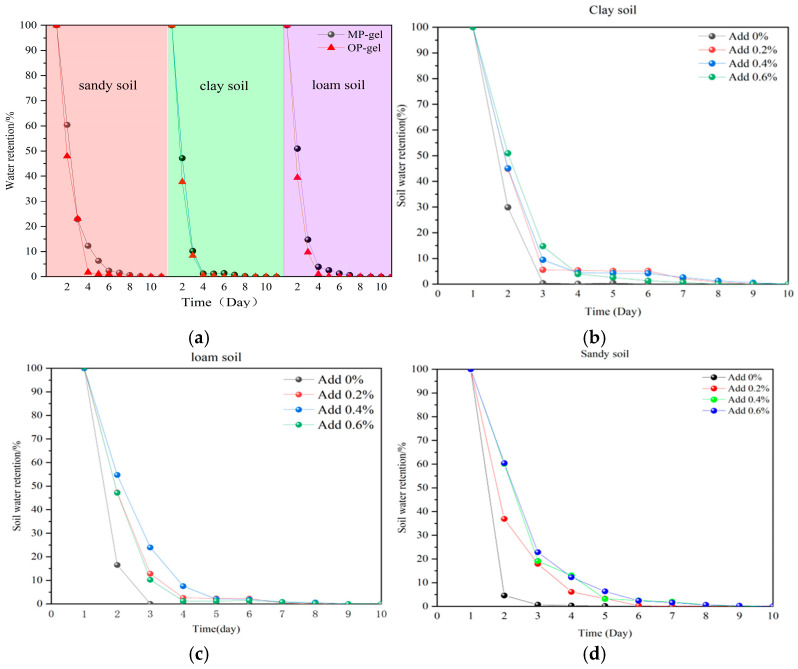
(**a**) Water retention capacity of MP-gel and OP-gel at 0.6% addition in different soils; (**b**–**d**) water retention capacity of different concentrations of MP-gels in different soils.

Based on the data presented in Figure 10a, the water retention performance of OP-gel in sand, loam, and clay soils was significantly inferior to that of MP-gel. Consequently, this study delved into a detailed investigation of MP-gel to ascertain its optimal concentration for use in different soil types. As depicted in Figure 10b–d, an addition of 0.2% MP-gel was found to significantly enhance the water retention of clay soil, extending the retention period from the third day to the eighth day. Furthermore, 0.4% MP-gel increased the water retention of loam soil by six days. It is worth noting that the increase in the amount of water gel added had the most significant effect on the water retention of the sandy soil, and the water retention was directly proportional to the amount of water gel added.

This study demonstrates that the addition of hydrogels can significantly enhance the water retention capacity of soil. The inherent characteristic of sandy soil is its relative looseness, which limits its water retention capacity [32]. The inclusion of MP-gel-4 can effectively fill the interstitial spaces between soil particles, thereby improving the soil’s water retention ability. In contrast, the addition of MP-gel-4 does not significantly enhance the water retention capacity of clay soil, as clay has smaller pores and inherently strong water retention properties [33]. Loamy soil, which lies between sandy and clay soils, is the most suitable type for crop growth [34]. The water retention effect of adding MP-gel-4 to loamy soil is intermediate between the other two soil types. It can be concluded that the addition of MP-gel-4 can significantly improve the water retention of all three soil types. However, to optimize resource utilization, the addition of hydrogels should be tailored to the specific soil type, as the addition of hydrogels does not necessarily lead to the most beneficial improvements.

The addition of MP-gel-4 to sandy soil resulted in a notable enhancement in water retention, particularly at a concentration of 0.6%. Conversely, the incorporation of MP-gel-4 into clay soil demonstrated a significant improvement in water retention at a relatively low concentration of 0.2%. The addition of further hydrogel does not enhance the water retention capacity of clay soil but rather diminishes the efficiency of hydrogel utilization. In loamy soils, the addition of 0.4% MP-gel-4 improves water retention and does not reduce the efficiency of hydrogel utilization.

### 2.7. Evaluation of MP-(AA-AAM)-Gel for Plant Growth Performance

The objective of this study was to investigate the impact of varying concentrations of hydrogel additions (0%, 0.5%, 1%, 1.5%, 2%, and 3%) on the growth of wheat seedlings subjected to drought-stress conditions. Wheat seedlings were cultivated under conditions of simulated drought, with their growth subjected to systematic monitoring to ascertain the role of hydrogels in enhancing the growth conditions of the seedlings.

**Figure 11:**
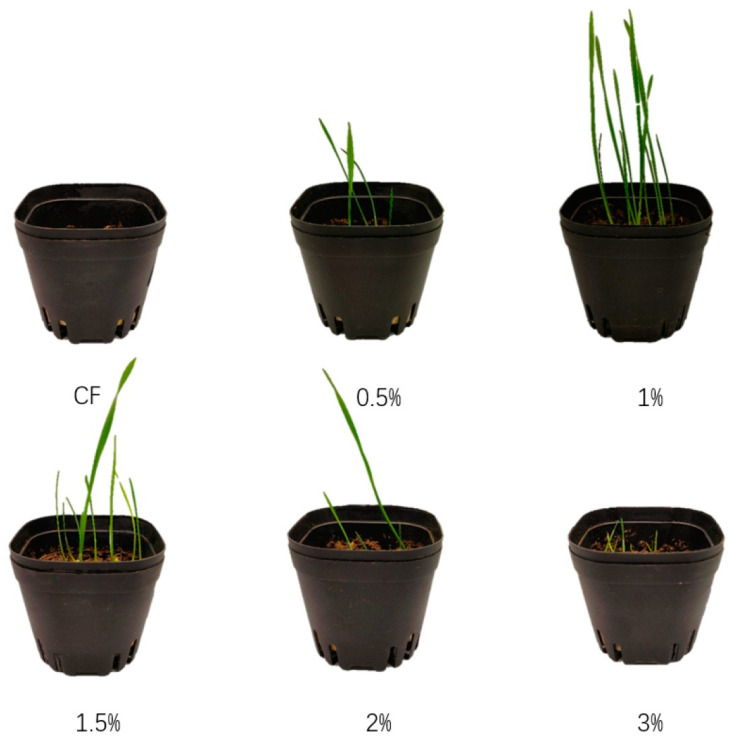
Growth of wheat seedlings in the second week under the addition of 0%, 0.5%, 1%, 1.5%, 2%, and 3% MP-gel.

From the fourth day of the experiment onwards, notable discrepancies were identified between the treatment groups with varying hydrogel additions. The growth of wheat seedlings in the absence of hydrogel (gel-4, 0%) and with 3% hydrogel (gel-4, 3%) exhibited a significantly inferior performance compared to that of the other treatment groups. On the 10th day, the wheat seedlings in the (gel-4, 0.5%) treatment group exhibited signs of wilting, while the seedlings in the other treatment groups continued to grow. As shown in Figure 11. On the 14th day, only the 1.5% and 2% treatments demonstrated the capacity for continued growth, while the other treatments exhibited signs of wilting and mortality among the wheat seedlings.

**Figure 12:**
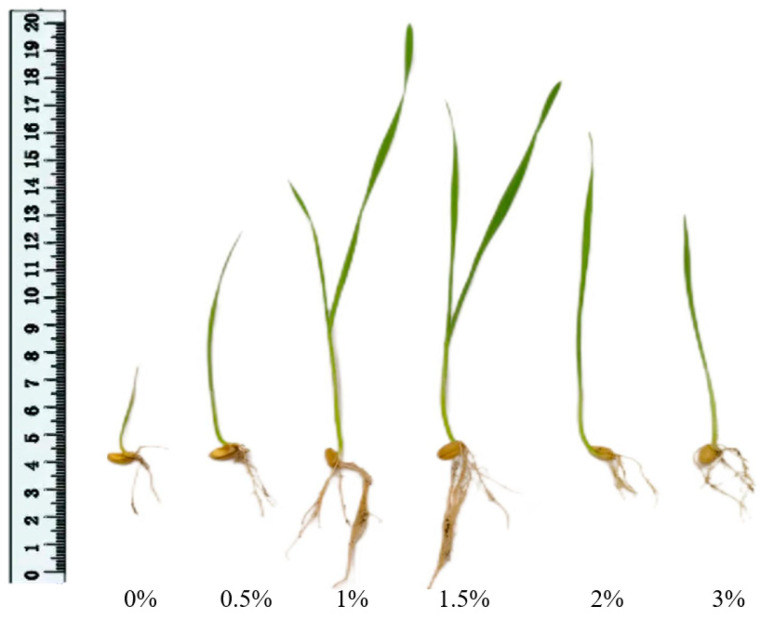
Seedling height of wheat after cessation of growth under 0%, 0.5%, 1%, 1.5%, 2%, and 3% MP-gel addition.

As shown in Figure 12 for the uprooted wheat seedlings, the plant heights of the treatment groups were compared. The results demonstrated that the (gel-4, 1.5%) treatment group exhibited the greatest wheat growth, followed by the (gel-4, 1%) treatment group, which exhibited a notable enhancement in wheat growth compared to the other treatment groups. In contrast, the treatment group lacking hydrogel (gel-4, 0%) and comprising 3% MP-gel-4 (gel-4, 3%) exhibited the poorest seedling growth.

This study has elucidated the positive role of hydrogels in enhancing crop growth under drought-stress conditions. The addition of an appropriate quantity of hydrogels significantly improves the soil’s water retention capacity, providing a stable water supply to crops, which is of great significance in water-scarce arid regions [35]. The experimental results indicate that the water retention capacity of hydrogels is closely related to their application ratio. An excessive quantity of hydrogels may not effectively supplement water and could even compete with crop seedlings for limited water resources, thus negatively affecting seedling growth. Therefore, determining the optimal hydrogel dosage is crucial for optimizing crop growth under drought conditions [36]. In this study, hydrogel addition ratios of 1.5–2% have been demonstrated to be the optimal proportions for promoting the growth of wheat seedlings. This finding provides practical guidance for agricultural production in arid regions, aiding in the enhancement of crop yield and water-use efficiency. Furthermore, the application of hydrogels can promote the utilization of agricultural waste materials and reduce reliance on traditional irrigation methods, which has positive implications for the sustainable development of agriculture. Future research could further explore the application effects of different types of hydrogels, as well as the optimal dosage under various crop and soil conditions, to provide more scientific and efficient solutions for agriculture in arid regions [37].

## 3. Conclusions

This study prepared the MP-gel and OP-gel series of composite hydrogels using MP and OP, respectively. The results of the comparison demonstrated that MP-gel exhibited superior performance in several key aspects, including water absorption, pH applicability range, reusability, soil water retention, and water-holding capacity. Furthermore, the application of an optimal amount of MP-gel was found to significantly enhance the growth of wheat seedlings under drought-stress conditions. This discovery provides a viable solution for crop cultivation and water management in arid regions.

## 4. Materials and Methods

### 4.1. Materials

MP and OP were purchased from the local market. The chemical reagents employed in this study were acrylic acid (AA), acrylamide (AAM), ammonium persulfate (APS), N, N′-methylene bisacrylamide (MBA), and sodium hydroxide (NaOH), which were manufactured by Tianjin Kemi Chemical Reagent Co. (Tianjin, China) and were of analytical purity. 

### 4.2. Preparation of MP/OP-(AA-AAM)-Gel

**Figure 13:**
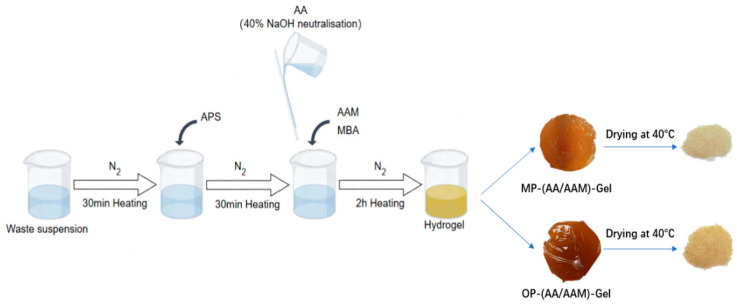
The process of hydrogel synthesis.

The synthesis of MP and OP grafted polymer hydrogels (MP-(AA-AAM)-gel and OP-(AA-AAM)-gel) is shown in Figure 13. A 7.5 wt% aqueous suspension of MP/OP was prepared by adding fresh MP/OP to water and homogenizing it using an IKA T50 digital disperser. A total of 384.0 g of the 7.5 wt% MP/OP suspension was added to a 1 L three-necked flask, which was equipped with a mechanical stirrer and a nitrogen line. The MP/OP suspension was purged with nitrogen for 30 min and subsequently heated under a nitrogen flow to a temperature of 70 °C. The initiator APS (1.152 g) was then introduced and the temperature was maintained at 70 °C. Following a 30 min interval, a predetermined quantity of AAM and AA (40% neutralized in aqueous NaOH) was introduced, along with the cross-linker MBA [38,39,40].

By varying the feed ratios of AA and AAM (from 7:3 to 3:7) and MBA (final concentration from 0.02 to 0.1 wt%), five MP-(AA-AAM)-gels, five OP-(AA-AAM)-gels, and one hydrogel product devoid of added peel waste were synthesized. The details of the feeds are presented in Table 1. The reaction was conducted at 70 °C under a nitrogen atmosphere.

The samples were dried in a dryer at 40 °C, ground to powder form, and sieved through a sieve of 0.5 mm pore size, and the sieved solid particles were used for subsequent experiments.

### 4.3. Methods of Characterization

#### 4.3.1. FTIR Spectroscopy

The Fourier Transform Infrared Spectrometer (FTIR) analysis was performed using a Thermo Fisher Nicolet iS50 instrument from Thermo Fisher Scientific (Shanghai, China). Samples were subjected to rapid compression at a pressure of 10–15 megapascals, and the FTIR spectrum of the powder was measured with a wavelength range of 400–4000 cm^−1^ [41].

#### 4.3.2. Morphological Characterization

The microstructure of the sample was studied using a Scanning Electron Microscope (SEM), specifically the JEOL S-3400N model from HITACHI (Tokyo, Japan). In this experiment, the electron acceleration voltage was stabilized at 8.0 kV to guarantee the image quality, and the gold plating method was used to enhance the contrast of SEM images [42].

#### 4.3.3. Swelling Study in Water

The dried gel powder (0.05 g) was weighed and transferred into pre-weighed tea bags that had been moistened in advance. Subsequently, the gel within the tea bags was soaked in tap water at room temperature for 24 h. The tea bags were then removed from the swelling medium and hung until no further water leaked out. The excess liquid was drained using filter paper and weighed in order to obtain the swelling weight of the hydrogel after 24 h of absorbing water. Additionally, the gel swelling ratio, *Q*, and the equilibrium swelling ratio, *Q_eq_*, were obtained using the following equation [43].

(1) $$Q_{eq}=\frac{W_{eq}-W_{0}}{W_{0}}$$

where *W* is the weight of the swollen sample, *W*_0_ is the weight of the dry sample, and *W_eq_* is the weight of the swollen sample after achieving equilibrium. The unit is g.

#### 4.3.4. Swelling Study in Different pH

The hydrogel was immersed in ultrapure water solutions, with the pH values adjusted from 3 to 12 using precise additions of hydrochloric acid or sodium hydroxide. The equilibrium swelling ratio (*Q_eq_*) of the gel was subsequently determined at each pH level using a pH meter to monitor the pH adjustments. Subsequently, the equilibrium swelling ratio (*Q_eq_*) of the hydrogels was plotted as a function of pH. A Gaussian distribution model was used to fit the pH value at which the samples reached their maximum water absorption ratio.

(2) $$y=y_{0}+\frac{A}{W\sqrt{\pi /2}}e^{-2\frac{{\left(x-x_{c}\right)}^{2}}{w^{2}}}$$

where *A* is the amplitude, *W* is the width, *x* is the pH, *y* is the maximum water absorption (unit is g/g), and *x_c_* and *y*_0_ are constants representing the center and baseline values of the function.

#### 4.3.5. Reusability

An amount of 50 mg of each sample was transferred into a pre-weighed sachet and the initial mass was recorded accurately. Subsequently, the samples were immersed in ultrapure water and allowed to stand for 24 h to reach swelling equilibrium. The mass was then measured in order to obtain the swelling ratio, designated as m1. Subsequently, the hydrogel samples that had reached equilibrium were transferred to a thermostat and maintained at 80 °C for a period of 6 h. This procedure ensures complete dehydration and allows for the assessment of the dehydration ratio and re-drying potential of the sample. To ensure the accuracy and reproducibility of the data, this comprehensive test protocol was repeated six times in succession [44].

#### 4.3.6. Water-Holding and Retention Studies in Different Soils

The hydrogel exhibiting superior performance in all respects was selected for evaluation in diverse soil types. The soil samples utilized in the experiment encompassed sandy soil, clay soil, and loam soil [45]. After obtaining soil samples, 30 g of soil was taken and mixed with dry hydrogel powder (*W_s_*) at 0.0%, 0.2%, 0.4%, and 0.6% relative to soil weight, followed by placement in pots containing tea bags (*W*_0_). The experimental materials (flower pot, tea bag, and mixed samples) were subsequently soaked in ultrapure water for one day. After 24 h, the material was removed and weighed as *W*_1_. Subsequently, the specimen was placed at room temperature and weighed daily to observe the changes, which were recorded as *W_t_*. Once a constant weight was reached, the dry weight was recorded as *W_dry_*. Water-holding (*W_h_*_%_) and water retention (*W_r_*_%_) images were plotted from the data [46]. The unit is g.

(3) $$W_{h\%}=\frac{W_{1}-W_{0}}{W_{s}}\times 100$$

(4) $$W_{r\%}=\frac{W_{t}-W_{dry}}{W_{1}-W_{dry}}\times 100\;$$

#### 4.3.7. Potting Trials

A total of 30 g of potting soil was weighed and subsequently amended with varying concentrations of hydrogel (0.0%, 0.5%, 1.0%, 1.5%, 1.5%, and 2.0%) for the cultivation of wheat seedlings. The wheat seedling seeds were rinsed three times with water and soaked for 7–10 h [47]. Following this, the seeds were whitened and planted into the potting soil with hydrogel added. The seeds were then placed in an incubator at a temperature of 23 °C and a humidity of 40%. Before germination, the seeds were sprayed with water daily to ensure sufficient moisture was available. Once germination had occurred, the process of spraying water was terminated, and the seedlings were removed from the incubator and relocated to an indoor setting. The growth of wheat seedlings was observed and recorded via photo records.

## Figures and Tables

**Table 1:** Materials for hydrogel synthesis.

No.	Mp/Op	APS	AAM	MBA	AA	NaOH	w
CK	0.0 g	1.152 g	17.28 g	0.288 g	40.32 g	8.96 g	124.00 g
gel-1	28.8 g	1.152 g	17.28 g	0.288 g	40.32 g	8.96 g	124.00 g
gel-2	28.8 g	1.152 g	17.28 g	0.144 g	40.32 g	8.96 g	124.14 g
gel-3	28.8 g	1.152 g	17.28 g	0.576 g	40.32 g	8.96 g	123.71 g
gel-4	28.8 g	1.152 g	28.80 g	0.288 g	28.80 g	6.40 g	126.56 g
gel-5	28.8 g	1.152 g	40.32 g	0.288 g	17.28 g	3.84 g	129.12 g

## Data Availability

Data sharing does not apply to this article as no datasets were generated or analyzed during the current study.
